# Contamination of nanoparticles by endotoxin: evaluation of different test methods

**DOI:** 10.1186/1743-8977-9-41

**Published:** 2012-11-09

**Authors:** Stijn Smulders, Jean-Pierre Kaiser, Stefano Zuin, Kirsten L Van Landuyt, Luana Golanski, Jeroen Vanoirbeek, Peter Wick, Peter HM Hoet

**Affiliations:** 1 Laboratory of Pneumology, Unit for Lung Toxicology, KU Leuven, Leuven, Belgium; 2 Empa, Swiss Federal Laboratories for Materials Science and Technology, Laboratory for Materials-Biology Interactions, St. Gallen, CH-9014, Switzerland; 3 Venice Research Consortium, c/o VEGA Park - Venice Gateway for Science and Technology, Venice, Italy; 4 KU Leuven BIOMAT, Department of Oral Health Sciences, KU Leuven, Leuven, Belgium; 5 CEA-Grenoble, Liten, Laboratory of Tracer Technologies, Grenoble, France

**Keywords:** Endotoxin, Nanoparticles, LAL assay, TLR4 reporter cells

## Abstract

**Background:**

Nanomaterials can be contaminated with endotoxin (lipopolysaccharides, LPS) during production or handling. In this study, we searched for a convenient *in vitro* method to evaluate endotoxin contamination in nanoparticle samples. We assessed the reliability of the commonly used limulus amebocyte lysate (LAL) assay and an alternative method based on toll-like receptor (TLR) 4 reporter cells when applied with particles (TiO_2_, Ag, CaCO_3_ and SiO_2_), or after extraction of the endotoxin as described in the ISO norm 29701.

**Results:**

Our results indicate that the gel clot LAL assay is easily disturbed in the presence of nanoparticles; and that the endotoxin extraction protocol is not suitable at high particle concentrations. The chromogenic-based LAL endotoxin detection systems (chromogenic LAL assay and Endosafe-PTS), and the TLR4 reporter cells were not significantly perturbed.

**Conclusion:**

We demonstrated that nanoparticles can interfere with endotoxin detection systems indicating that a convenient test method must be chosen before assessing endotoxin contamination in nanoparticle samples.

## Background

Nanoparticles are worldwide produced and used in various commercially available applications (cosmetics, paints, textiles) [1] and predictions estimate that by 2014, more than 15% of all products on the global market will have some kind of nanotechnology incorporated into their manufacturing process [2]. Besides their ubiquitous lucrative effects, also toxic effects have been reported [3]. It cannot be excluded that nanoparticles, especially when they were not kept sterile, can be contaminated with endotoxin during production or handling. Therefore, endotoxin contamination should be assessed when evaluating the potential toxicity, to distinguish specific nanoparticles toxicity from the endotoxin effects.

Endotoxins or lipopolysaccharides (LPS) are large (molecular weight: 200 to 1000 kDa), heat-stable (to 100°C) molecules that form the major structural components of the outer cell wall of gram-negative bacteria [4,5]. High levels of endotoxin are omnipresent in our living environment, and exposure can induce a variety of biological effects such as airway disease, fever, hypotension, coagulopathies, septic shock and even death. Endotoxin consists of a bioactive lipid component, termed lipid A, covalently bound to a hydrophilic heteropolysaccharide of variable length [6]. Induction of a signal transduction cascade evolves binding of endotoxin on CD14 followed by association with the protein MD2 and the transmembrane TLR4 [7]. This finally results in the release of inflammatory cytokines, including IL-1β, TNF-α and IL-6, mainly secreted by immune cells such as macrophages and dendritic cells.

Currently, the LAL test is the assay of choice for the determination of endotoxin in medicines, biological products and medical devices [5]. In general, three different LAL assays are used worldwide: gel clot, turbidimetric (increase in turbidity) and chromogenic (color formation) assay. A good overview of the different assays, along with their advantages and disadvantages, can be found in the review article of Hurley [5].

In spite of the new ISO norm published in 2010 on endotoxin test on nanomaterial samples for *in vitro* systems [8], not much is known in which way nanoparticles interfere with the different types of LAL assays [9]. The aim of our study is to find a convenient *in vitro* test method to evaluate endotoxin contamination in nanoparticle samples. Therefore, in this study, we assessed the reliability of a gel clot LAL assay, an endpoint chromogenic LAL assay and a FDA-licensed endotoxin detection system when performed in the presence of nanoparticles, as well as the proposed sample preparation methods of the ISO norm were evaluated. Moreover, as an alternative for the LAL assay, we tested another method based on TLR4 reporter cells to measure endotoxin in nanoparticle formulations.

## Results

**Figure 1:**
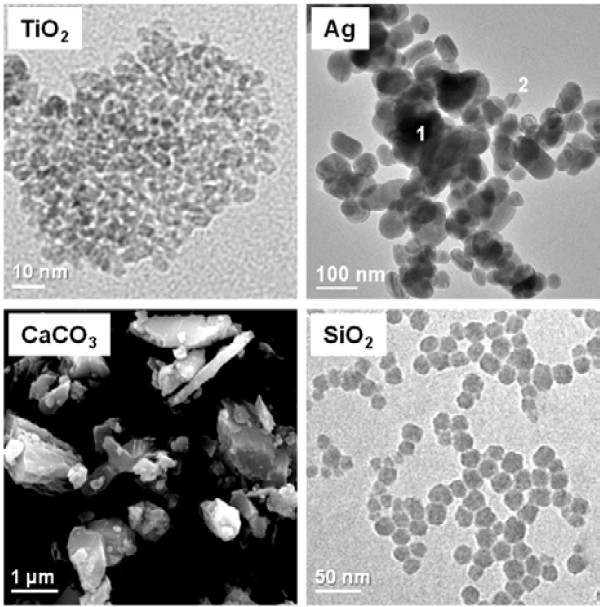
**Electron microscopy images of the different particles.** Images of TiO_2_, Ag and SiO_2_ particles were obtained by transmission electron microscopy (TEM). CaCO_3_ image was obtained by scanning electron microscopy (SEM).

Characteristics of TiO_2_, Ag, CaCO_3_ and SiO_2_ particles are summarized in Table 1, electron microscopy images are shown in Figure 1. Transmission electron microscopy (TEM) analysis showed average particle sizes of 15 nm (TiO_2_), 25 to 85 nm (Ag), and 19 nm (SiO_2_). Scanning electron microscopy (SEM) analysis of CaCO_3_ revealed a very heterogeneous composition, showing particles of both nano- and micrometer sizes. Analysis of the particles by dynamic light scattering (DLS) showed single populations of 396 nm (TiO_2_), 90 nm (Ag) and 192 nm (SiO_2_), and two populations of 67 and 582 nm in the CaCO_3_ sample. All particles were negatively charged, the largest electrostatic stabilization was found in the Ag and SiO_2_ samples showing zeta potentials of respectively −42 and −40 mV.

**Table 1:** **Characteristics of TiO**_**2**_**, Ag, CaCO**_**3 **_**and SiO**_**2 **_**particles**

	**TiO**_**2**_	**Ag**	**CaCO**_**3**_	**SiO**_**2**_
Average TEM or SEM size (nm)	15 ± 4	From 25 (spherical) to 80 – 90 nm (rods)	From nano- to micrometer size	19 ± 4
Hydrodynamic diameter (nm)	396 (P10: 164, P90: 1128)	90 (P10: 52, P90: 121)	67 (P10: 41, P90: 139) and 582 (P10: 267, P90: 1361	192 (P10: 105, P90: 332)
Zeta potential (mV)	- 25 ± 1	- 42 ± 0.8	- 14 ± 0.5	- 40 ± 1.1
Shape	Almost spherical	Some spherical, others rods	Mixed, flakes	Spherical

TEM/SEM size, hydrodynamic diameter, zeta potential and shape of the different particles. TEM/SEM size and zeta potential are expressed as mean ± SD. Hydrodynamic diameter is expressed as an intensity-weighted average with P10 (probability 10%) and P90 (probability 90%).

**Figure 2:**
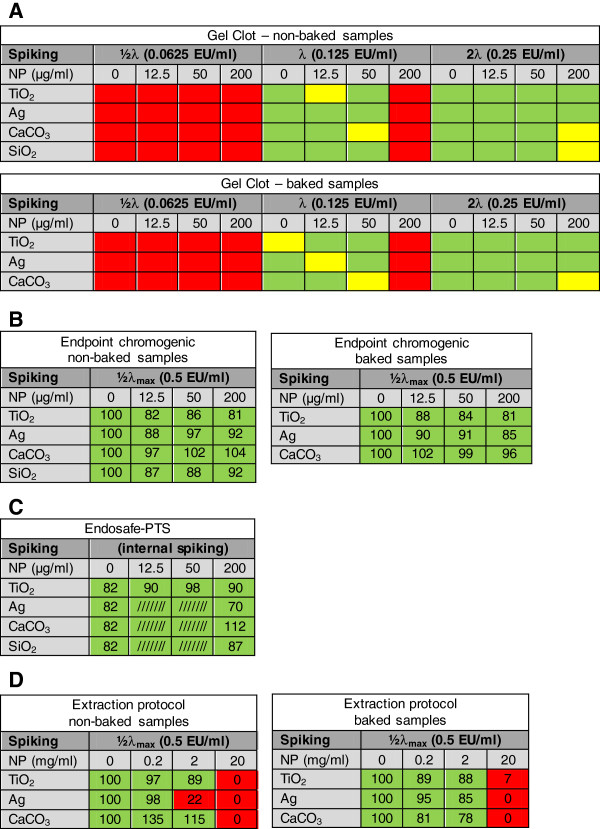
**Overview of the different LAL assays when performed in the presence of particles (12.5, 50 and 200 μg/ml or 0.2, 2 and 20 mg/ml) after spiking.A**: Gel clot LAL assay; **B**: Endpoint chromogenic LAL assay; **C**: Endosafe-Portable Test System (PTS); **D**: Endpoint chromogenic after endotoxin extraction (shaken 10 min and centrifugation). Samples spiked with endotoxin concentrations half of assay sensitivity (½λ: 0.0625 EU/ml), assay sensitivity (λ: 0.125 EU/ml) and double of assay sensitivity (2λ: 0.25 EU/ml) (gel clot LAL assay); half of maximum of assay range (½λ_max:_ 0.5 EU/ml) (endpoint chromogenic LAL assay); the endosafe-PTS contains an internal spiking control. Results are shown in a heat plot: Red: no clot (Gel Clot) or no spike recovery (chromogenic assays); Yellow: increased turbidity (Gel Clot); Green: clot formation (Gel Clot) or complete spike recovery (chromogenic assays). Percentages indicate percentage spike recovery. In case of the endosafe-PTS, measurements of Ag, CaCO_3_ and SiO_2_ samples were not performed at the lower particle concentrations (12.5 and 50 μg/ml), because the highest concentration (200 μg/ml) showed a complete spike recovery.

The results of the spiking experiments of the different LAL assays (gel clot, endpoint chromogenic, endosafe-Portable Test System (PTS) and endotoxin extraction protocol) are summarized in Figure 2.

In the gel clot LAL assay, negative results (no clot formation) were obtained with all particles at each concentration (12.5, 50 and 200 μg/ml) both for non-baked and baked particle samples (negative controls) (data not shown). In the presence of non-baked particles, spiking at half of assay sensitivity (½λ: 0.0625 EU/ml) resulted not in clot formation (negative result) at all particle concentrations (Figure 2A); spiking at assay sensitivity (λ: 0.125 EU/ml) leads not to clot formation at the highest particle concentration (200 μg/ml), while no inhibition of clot formation was seen at lower concentrations (12.5 and 50 μg/ml). After spiking non-baked particles at double of assay sensitivity (2λ: 0.25 EU/ml) no inhibition of clot formation was observed for the TiO_2_ and Ag samples, whereas only an increased turbidity, but no clot, was observed in CaCO_3_ and SiO_2_ at 200 μg/ml. The same results were observed for baked spiked TiO_2_, Ag and CaCO_3_ samples.

**Figure 3:**
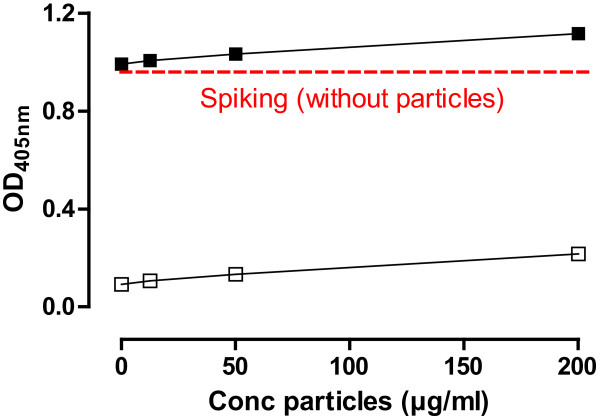
**Optical density by increasing concentrations of TiO**_**2 **_**particles in the endpoint chromogenic LAL assay.** Open symbols show optical density (OD_405nm_) of TiO_2_ particles, while closed symbols show the OD of spiked TiO_2_ particles. The dotted line shows the corrected OD after spiking: OD(spiked particles) – OD(particles).

In all samples (baked and non-baked), no (or at least lower than the detection limit) endotoxin contamination was measured in the endpoint chromogenic LAL assay (data not shown). A complete spike recovery was seen for all particle samples (non-baked and baked) at all applied concentrations (Figure 2B). Figure 3 shows the gradual increase of background optical density by increasing concentrations of TiO_2_ particles. A similar increase was seen in case of the Ag particles (data not shown). The background particle optical density was substracted from the corresponding measured values in the endpoint chromogenic LAL assay to obtain the final results. In the endosafe-PTS, all particle samples showed a complete spike recovery (within 50-200% tolerance limits) at all concentrations (Figure 2C). In all non-spiked samples, no (or at least lower than the detection limit) endotoxin contamination was measured in the endosafe-PTS LAL assay (data not shown).

The endotoxin extraction procedure was performed, as described in the ISO protocol on endotoxin tests on nanomaterial samples with the aim to reduce particle assay interference. Therefore, the endotoxin concentration was measured in the supernatant after centrifugation with the endpoint chromogenic LAL assay. TiO_2_, Ag and CaCO_3_ samples (baked and non-baked) tested negative at all applied concentrations (0.2, 2 and 20 mg/ml) (data not shown). After spiking (0.5 EU/ml), no endotoxin could be recovered at the highest particle concentration (20 mg/ml) for all particles (baked and non-baked) (Figure 2D). At lower particle concentrations (0.2 and 2 mg/ml), the spike recovery was within the tolerance range of 50-200% for all particles (baked and non-baked), except for the non-baked Ag sample (2 mg/ml) in which we only measured a spike recovery of 22%.

**Figure 4:**
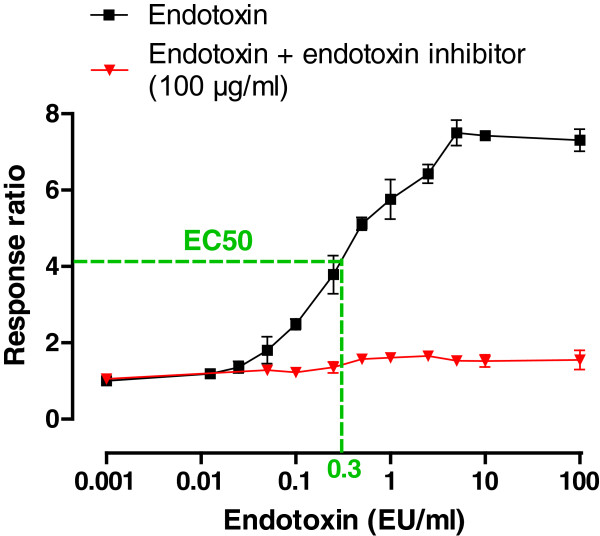
**Dose–response curve of TLR4 reporter cells without and with endotoxin inhibitor.** Black line shows response ratio of TLR4 reporter cells by increasing concentrations of endotoxin. Red line shows response ratio when applied with endotoxin inhibitor (polymyxin B sulfate, 100 μg/ml). EC_50_: Half maximum effective concentration.

TLR4 reporter cells were used to evaluate the influence of the particles on TLR4-activation by endotoxin. Figure 4 shows the responsiveness of the cells to increasing concentrations of endotoxin. TLR4 reporter cells already show an increased response starting at an endotoxin concentration of 0.05 EU/ml. Assuming this dose–response relationship, the half maximum effective concentration (EC_50_) was determined (0.3 EU/ml), causing a response ratio of about 4, and this concentration was chosen for spiking (½λ_max_). Also the biological functionality (TNF-α release) of the cells was assessed. No increase in TNF-α release was observed after exposure to endotoxin (See in Additional file 1: Figure S1).

**Figure 5:**
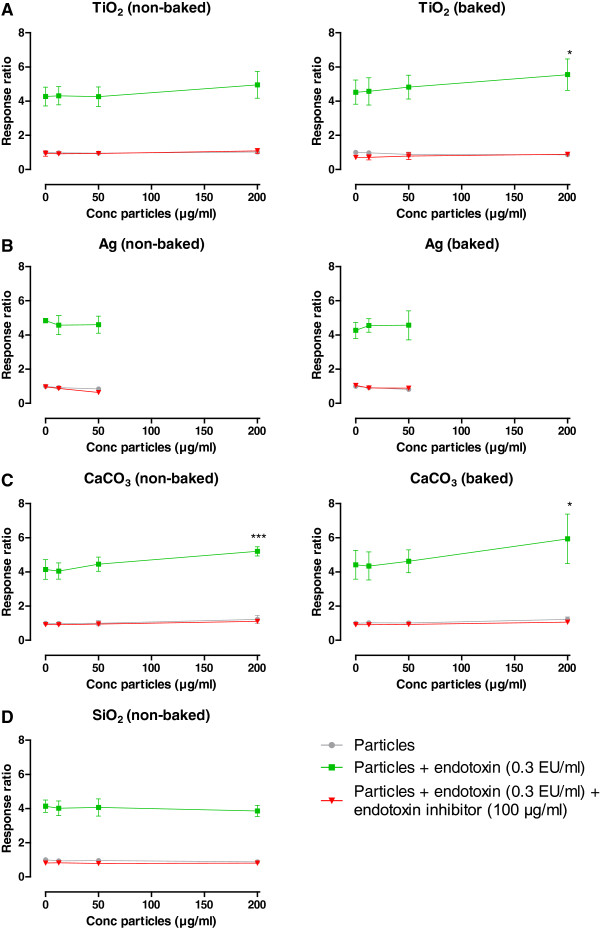
**Response ratio of TLR4 reporter cells when applied with particles.** The response ratio of TLR4 reporter cells after exposure to increasing concentration of particles (0, 12.5, 50 and 200 μg/ml) (grey line), spiked (0.3 EU/ml) particles (green line) and spiked particles + endotoxin inhibitor (polymyxin B sulfate, 100 μg/ml) (red line). **A**: TiO_2_ particles (non-baked and baked); **B**: CaCO_3_ particles (non-baked and baked); **C**: Ag particles (non-baked and baked); **D**: SiO_2_ particles (non-baked).

**Figure 6:**
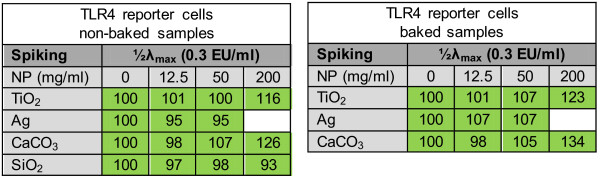
**TLR4 reporter cells when performed in the presence of particles (12.5, 50 and 200 μg/ml) after spiking.** Samples spiked with endotoxin concentrations half of assay sensitivity (½λmax: 0.3 EU/ml). Results are shown in a heat plot: Red: no spike recovery; Green: complete spike recovery (between 50 and 200%). Percentages indicate percentage spike recovery.

Exposing the cells to the particles (non-baked and baked), did not result in a measurable response with increasing concentrations of particles in all cases (Figure 5 (grey lines)). Spiking (½λ_max_) the particles (positive control) resulted in a higher response compared to the respective non-spiked samples at all particle concentrations and approximates the response observed at the endotoxin EC_50_ value (response ratio of approximately 4), indicating a complete spike recovery (Figure 5 (green lines) and Figure 6). Moreover, an increased response was seen in the spiked TiO_2_ and CaCO_3_ samples at a concentration of 200 μg/ml compared to the respective spiked sample without particles, which was significant in the case of TiO_2_ (baked) and CaCO_3_ (non-baked and baked). Applying an endotoxin inhibitor (polymyxin B sulfate, 100 μg/ml) in the presence of spiked particles resulted in a complete inhibition of the response at all particle concentrations (Figure 5 (red lines)). Regarding Ag, significant cytotoxicity was observed at concentrations above 50 μg/ml and therefore, measurements were limited in this case.

## Discussion

In the present study, we investigated the performance of different LAL assays in the presence of nanoparticles and evaluated another – non LAL-based – *in vitro* assay to assess endotoxin contamination. We have shown that nanoparticles have the potential to interfere with the gel clot LAL assay at elevated – but not excessive high – particle concentrations. Likewise, an endotoxin extraction protocol, including shaking and centrifugation of the particle dispersion, seems to be unsatisfactory at high particle concentrations. Furthermore, we also demonstrated that chromogenic-based LAL endotoxin detection systems (chromogenic LAL assay and Endosafe-PTS) and TLR4 reporter cells report no interfering effects at all applied particle concentrations.

Over the past decades, the LAL assay has been found an application in various domains, ranging from the pharmaceutical and aerospace industry [10] to toxicological research. The potential interference of biological products has been extensively studied and it is known that certain body fluids (e.g. urine and blood) can influence the outcome of the LAL assay [5,11]. A study carried out by the FDA almost 30 years ago showed that out of the 333 drug products tested, 236 (71%) interfered with the LAL assay when applied prior to any dilution [12]. Surprisingly, almost no research has been done to which extent nanoparticles can interfere with the different LAL assays. Recently, Dobrovolskaia *et al.* published the first study showing that endotoxin levels can be under- or overestimated due to the presence of nanoparticles [9].

Interference can occur when endotoxin interferes with the particles or when particles interfere with the LAL specific enzymes resulting in the decrease or increase of the sensitivity of the assay. Therefore, appropriate inhibition/enhancement controls are essential to recognize whether negative results are due to absence of endotoxin, or inhibition of the assay. According to the United States, European and Japanese pharmacopeia, a test is considered valid if the measured concentration of endotoxin added falls within the tolerance range of 50-200% of the known added endotoxin concentration [13-15]. Appropriate spiking concentrations need to be chosen dependent on the applied LAL assay and its associated sensitivity.

The gel clot LAL assay is quite easy to perform, but limitations are the subjective endpoint and the relative lack of sensitivity. In our study, endotoxin spiking concentrations were chosen below (50% assay sensitivity; ½λ: 0.0625 EU/ml), at (100% assay sensitivity; λ: 0.125 EU/ml) and above (200% assay sensitivity; 2λ: 0.25 EU/ml) the sensitivity of the assay. In this way, we were able to assess whether potential interference was situated in- or outside the tolerable limits. Two of four tested particles, SiO_2_ and CaCO_3_, fall out of this range and exert substantial inhibitory effect on the gel clot LAL assay, at the highest particle concentration tested (200 μg/ml). However, the other tested particles, TiO_2_ and Ag, show inhibitory effects as well, but only at spiking concentrations at the sensitivity of the assay (λ), and fall therefore within the 50-200% limits. Similarly, in the study of Dobrovolskaia *et al.*, 3 of the 5 tested particle formulation interfered with the gel clot LAL assay [9]. Dilution is the simplest and most widely used technique to overcome interference, in our experiments no more inhibitory effects were seen after diluting to particle concentrations of 50 and 12.5 μg/ml. Important to note is that particle concentrations of about 200 μg/ml are often used in toxicological research [16,17], thus adequate endotoxin detection methods at this concentration are indispensable.

The chromogenic-based LAL assays (chromogenic LAL assay and the Endosafe-PTS) are, compared to the semiquantitative gel clot LAL assay, more quantitative. Neither in the chromogenic LAL assay, nor in the Endosafe-PTS, any significant interfering effect due to the presence of the particles were observed at the highest applied concentration (200 μg/ml), suggesting that chromogenic-based LAL methods are convenient for endotoxin testing in particle samples. However, at increasing concentrations of certain particles, in our study in particular the TiO_2_ and Ag particles, a growing background color/optical density has to be taken in account. The increase of optical density, caused by the particles, could therefore be misinterpreted as increasing levels of endotoxin. Oostingh *et al.* reached the same conclusion regarding the use of spectophotometric detection techniques in the case of nanoparticle samples [18]. They showed that Au nanoparticles, already at very low concentrations (starting from 1 μg/ml), significantly interfered with the spectophotometric readout in the endpoint chromogenic LAL assay. Therefore, background optical density should always be substracted from their respective measured value. At too high particle concentrations, measurements can become unreliable and in this case another endotoxin detection method needs to be considered.

In 2010, ISO published an international standard on endotoxin test on nanomaterial samples for *in vitro* systems (LAL test), including an endotoxin extraction method [8]. Comparable procedures are already performed to extract endotoxin from air filters and dust particles [19,20]. Usually, water is the extraction medium, other media like polysorbate 20 can be used but these can interfere with the LAL assay. As mentioned above, particles can interfere with spectophotometric detection methods and therefore, in the endotoxin extraction experiments, we chose to work with higher particle concentrations (up to 20 mg/ml). In our study, extraction in water lead to a complete spike recovery at the two lowest particle concentrations (0.2 and 2 mg/ml) in most samples, suggesting this procedure is adequate for many nanoparticle suspensions. However, at very high particle concentrations (20 mg/ml) no endotoxin could be recovered indicating the limitations of this technique. Furthermore, particles that do not pellet during classical centrifugation are not suitable for this method.

Knowing that nanoparticles are coated with proteins when entering a biological fluid (e.g. blood, plasma) [21], it cannot be excluded that endotoxin and/or LAL proteins bind on the particle surface, possibly resulting in modification/inactivation of those attached molecules. However, as demonstrated interfering effects were only seen in the gel clot LAL assay, not in the chromogenic-based LAL assays, suggesting gel clot specific proteins are probably involved in the inhibitory effects. We hypothesize that interaction between the particles and the coagulogen protein or its activated counterpart (coagulin) causes the observed inhibitory effects in the gel clot LAL assay. Likewise, the unsuitability of the endotoxin extraction protocol at high particle concentrations, in our hand above 2 mg/ml, can be attributed to the attached endotoxin does not wash off during shaking or (a part of) the free endotoxin ends up in the pellet, captured in a matrix of particles, after centrifugation.

The lipid A component represents the toxic and immunomodulating domain of endotoxin and it is the part of the molecule that is reactive in both the LAL assay and *in vivo* after binding on the TLR4 receptor [7]. TLR4 reporter cells are widely used to screen and validate TLR4 agonists and antagonists. To our knowledge, we are the first that used TLR4 reporter cells as a tool to evaluate endotoxin contamination, and the potential interference of nanoparticles. We observed a complete spike recovery in all samples when exposing the TLR4 reporter cells to increasing concentration of spiked particles, indicating that TLR4 reporter cells can be used as a replacement for the commonly used LAL assay. Because TLR reporter cells already show an increased response starting at an endotoxin concentration of 0.05 EU/ml, the sensitivity is comparable to those of the LAL assay. However, nanoparticles can exert cytotoxic effects on the TLR4 reporter cells, limiting the measurements above particle cytotoxicity.

Furthermore, an increased response was seen in the spiked TiO_2_ (baked) and CaCO_3_ (non-baked and baked) samples at the highest concentration compared to the respective spiked samples without particles. We reasoned that sedimentation of the particles on the cells during incubation results in higher concentrations of particles, accompanied by higher concentrations of endotoxin bound on the particles and/or endotoxin captured in the matrix of the particles, in close proximity of the cells. Recently, Cho *et al.* demonstrated that particles can sediment, which means that the concentrations of particles on the cell surface at the bottom of a culture plate may be higher than the initial bulk concentration, and this could lead to increased activation or uptake by cells [22].

To produce LAL assays, horseshoe crabs are caught, bled and then returned to the ocean alive. Throughout this process, the crabs are exposed to a variety of potential stressors, such as air exposure, increased temperature, handling and blood loss [23]. Mortality associated with the collection and bleeding procedures may not be neglected, several studies have estimated mortality rates between 8 and 20% [23-26]. From an economic and ethical perspective, alternative endotoxin detection methods should be considered if available. From this point of view, TLR4 reporter cells can potentially be used as an alternative for the commonly used LAL assay, however, drawbacks are potential cytotoxicity and the relative long measurement time.

## Conclusion

In conclusion, our results indicate that nanoparticles (TiO_2_, Ag, CaCO_3_, SiO_2_) can interfere with certain endotoxin detection methods (gel clot LAL assay, endotoxin extraction protocol), while other assays (chromogenic-based LAL assay, TLR4 reporter cells) are not hampered. Dependent on the particle and its concentration used, a convenient endotoxin detection test method must be chosen.

## Methods

### Materials

Ag and CaCO_3_ powders were provided by PPG Europe BV (The Netherlands), while TiO_2_ powder and SiO_2_, in suspension at a concentration of 370 mg/ml, were obtained from respectively Materis Paints Italia (Italy) and Akzo Nobel Coatings S.A. (The Netherlands). The gel clot PYROGENT® Plus LAL assay and the endpoint chromogenic QCL-1000® LAL assay were purchased from Lonza (Verviers, Belgium). The Endosafe®-Portable Test System (PTS) Cartridges were purchased from Charles River (Wilmington, United States). HEK-Blue^TM^ hTLR4 (TLR4 reporter) cells, QUANTI-Blue^TM^ and 250X HEK-Blue^TM^ Selection were obtained from Invivogen (Toulouse, France), while endotoxin inhibitor (polymyxin B Sulfate) was purchased from Calbiochem (Darmstadt, Germany). Dulbecco’s Modified Eagle Medium (DMEM), fetal bovine serum (FBS), penicillin–streptomycin (10,000 U/ml and 10,000 μg/ml), fungizone, L-glutamine (200 mM) were purchased from Invitrogen (Merelbeke, Belgium). Endotoxin (E. coli strain O111:B4) was purchased from Sigma Aldrich (Bornem, Belgium).

### Particle characterization

#### DLS

TiO_2_, Ag, CaCO_3_ and SiO_2_ particles were diluted in water to concentrations of respectively 8, 20, 40 and 400 μg/ml. DLS measurements were performed with a Brookhaven 90 Plus NanoParticle Size Distribution Analyser (scattering angle 90 u, wavelength 659 nm, power 15 mW; Brookhaven Instruments Ltd, Redditch, UK). Correlation functions were analysed using the Clementine package (maximum entropy method) for Igor Pro 6.02A (WaveMetrics, Portland, OR, USA). This resulted in intensity-weighted distribution functions versus decay times. By converting the decay times with instrument parameters and physical parameters to hydrodynamic diameters, an intensity-weighted size distribution is obtained. A log-normal fit was applied to each population, resulting in the intensity-weighted average hydrodynamic diameter of the population.

#### Zeta potential

The zeta potential was measured in distilled water using a Brookhaven 90Plus/ZetaPlus instrument applying electrophoretic light scattering. A primary and reference beam (659 nm, 35 mW), modulated optics and a dip-in electrode system were used. The frequency shift of scattered light (relative to the reference beam) from a charged particle moving in an electric field is related to the electrophoretic mobility of the particle. The Smoluchowski limit was used to calculate the zeta potential from the electrophoretic mobility.

#### TEM

Suspensions of the SiO_2_ particles were applied on formvar-coated cupper mesh grids. After drying overnight, the particles were characterized by transmission electron microscopy (TEM) (JEOL JEM-1200 EX-II, Tokyo, Japan) at a magnification of 20.000-200.000 x. TiO_2_ and Ag pristine particles were suspended in ethanol, and a 5 μl drop of these dispersions were then deposited on a holey carbon film supported on 3 mm copper grids for TEM investigations. After solvent evaporating at room temperature, grids were dried overnight at dark at 25°C. Size and shape of particles were determined by using JEOL JEM - 3010 TEM, operating at 300 kV, with a high-resolution pole piece (0.17 nm point to point resolution) and equipped with Energy Dispersive X-ray Spectroscopy (EDS) detector (Oxford Link ISIS).

#### SEM

The CaCO_3_ powder was applied on aluminum stubs covered with self-adhesive carbon tabs (G3347N, Agar Scientific, Essex, UK) and was subsequently characterized by SEM (Jeol JSM-6610 LV).

### General strategy

To cover a broad spectrum of *in vitro* endotoxin detection methods, different types of LAL assays - gel clot, endpoint chromogenic and kinetic chromogenic (Endosafe-PTS) - were used to evaluate the potential endotoxin contamination in the particle samples. In addition to the LAL assay, a cell-based method (TLR4 reporter cells) was tested on its potential to detect endotoxin in the presence of particles.

The powder samples (TiO_2_, Ag and CaCO_3_) were baked for 4 h at 200°C to remove all endotoxin (negative controls).

To study the effect of the particles on the endotoxin detection, the samples (baked and non-baked) were spiked with different amounts of endotoxin in the different assays (positive controls).

### Sample preparation

Powder samples (TiO_2_, Ag and CaCO_3_) were prepared by suspending the particles in endotoxin-free water and a dilution series (0, 12.5, 50 and 200 μg/ml) was made in case of the gel clot LAL assay, endpoint chromogenic LAL assay, the Endosafe-PTS LAL assay and the experiments with TLR4 reporter cells. Dilutions of the SiO_2_ suspension were prepared to reach equal concentrations.

In addition, sample suspensions were prepared at concentrations of 0.2, 2 and 20 mg/ml and subsequently vortexed thoroughly, shaken (10 min) and centrifuged (2 min, 1000 g) to extract endotoxin, according to the ISO protocol (ISO 29701) [8]. The supernatant served as sample in the endpoint chromogenic LAL assay. In two preliminary tests, we verified whether endotoxins will pellet in particle free samples, possibly resulting in an underestimation of the contamination, and whether particles pellet during centrifugation. This clearly showed that endotoxin in suspension will not simply pellet and remained easily detectable (data not shown). TiO_2_, Ag and CaCO_3_ clearly pellet during centrifugation, while SiO_2_ particles remained in suspension during centrifugation (see in Additional file 1: Figure S2), and therefore no endotoxin extraction experiments were performed with SiO_2_ particles.

### LAL assay

The LAL assay is based on clottable proteins present in the blood cells (amebocytes) of the horseshoe crab (*Limulus polyphemus*) as described by Levin and Bang [27-29].

#### Gel Clot assay

In the gel clot LAL assay, activation of a preclotting enzyme cleaves the coagulogen protein to form a gelatinous clot. LAL reagent is added to an equal volume of sample and the formation of a clot is determined. The sensitivity of the gel clot LAL assay we used was 0.125 EU/ml, thus samples were spiked with endotoxin (*E. coli* strain O55:B5) concentrations half of assay sensitivity (½λ: 0.0625 EU/ml), assay sensitivity (λ: 0.125 EU/ml) and double of assay sensitivity (2λ: 0.25 EU/ml). Measurements were performed according to the manufacturer’s instructions.

#### Endpoint chromogenic LAL assay

In the chromogenic LAL assay, the coagulogen protein is replaced by a chromogenic substrate, a small peptide linked to a chromophore (p-nitroaniline) containing amino acid sequence, which can be cleaved by the clotting enzyme. The (yellow) color generated by cleavage of the substrate, as measured spectrophotometrically at 405 nm, is proportional to the amount of endotoxin in the sample. In the endpoint chromogenic LAL assay, the endotoxin concentration is measured once after a fixed time. The detection range was from 0.1 to 1.0 EU/ml, samples were spiked with an endotoxin (*E. coli* strain O111:B4) concentration in between this range (½λ_max_: 0.5 EU/ml). Measurements were performed according to the manufacturer’s instructions.

#### Endosafe-PTS

The Endosafe-PTS LAL assay is a FDA-licensed endotoxin detection system. The LAL assay cartridges contain four channels to which LAL reagent and a chromogenic substrate have been applied. Two of the four channels contain an endotoxin spike and serve as the positive control. Readouts between 50% and 200% spike recovery are considered to be acceptable. The sensitivity of the assay we used was 0.05 EU/ml.

### TLR4 reporter cells

TLR4 reporter cells were obtained by co-transfection of the human TLR4 (hTLR4) and MD-2/CD14 co-receptor genes and an optimized secrected embryonic alkaline phosphatase (SEAP) reporter gene under the control of a promoter (IL-12 p40) inducible by the transcription factors NF-ĸB and activator protein 1 (AP-1). TLR4 stimulation causes SEAP production, which can be easily determined spectophotometrically by QUANTI-Blue^TM^. TLR4 reporter cells were grown in DMEM supplemented with 5% (v/v) FBS, 100 U/ml penicillin, 100 μg/ml streptomycin, 1.25 μg/ml fungizone, 2 mM L-glutamine and 1X HEK-Blue^TM^ Selection.

HEK-TLR4 cells were exposed to a dilution series of endotoxin (*E. coli* strain O111:B4) to generate a dose response curve, the half maximum effective concentration (EC_50_) was determined which served as spiking concentration (½λ_max_). Measurements were performed according to the manufacturer’s instructions. Shortly, each sample was added to ~25,000 cells in a flat-bottom 96- well plate. After incubation for 22 h (at 37°C, 5% CO_2_), the cell supernatant was added to QUANTI-Blue^TM^. After incubation for 2 h, SEAP levels were measured spectophotometrically at 655 nm.

In preliminary experiments, we assessed the cytotoxicity of TLR4 reporter cells after exposure to polymyxin B Sulfate (100 μg/ml) or nanoparticles. Polymyxin B sulfate, TiO_2_, SiO_2_ and CaCO_3_ did not cause significant cell death, while cytotoxicity was observed after Ag exposure only at highest concentration (200 μg/ml).

### Data reporting - presentation

According to the United States, European and Japanese pharmacopeia, a test is considered valid if the measured concentration of endotoxin added falls within the tolerance range of 50-200% of the known added endotoxin concentration [13-15].

## Competing interests

None of the authors have competing interests.

## Authors’ contributions

SS, JPK, JV, PW, LG and PHMH were involved in setting up the experiments; SZ and KLVL were involved in the strategy to characterize correctly the materials in view of the research question. SS and JPK performed the experiments assessing endotoxin levels, along with writing the manuscript. KLVL and SZ performed particle characterization including TEM imaging and thoroughly read the manuscript. PHMH and JV are the supervisors of SS and contributed to the study design and helped to draft the manuscript. PW, the supervisor of JPK, contributed to the study design and thoroughly read the manuscript. All authors read and approved the final manuscript.

## Supplementary Material

Additional file 1 **Figure S1.** TNF-α release of TLR4 reporter cells after exposure to different concentrations of endotoxin. **Figure S2.** Pictures of particle samples (20 mg/ml) after centrifugation (2 min, 1000 g). Click here for file
