# Supplementary material for: Contamination of nanoparticles by endotoxin: evaluation of different test methods
Source: Part Fibre Toxicol. 2012 Nov 9;9:41. doi: 10.1186/1743-8977-9-41 (PMC3546036; doi:10.1186/1743-8977-9-41)

**Additional file**

**Contamination of nanoparticles by endotoxin: evaluation of different test methods**

Stijn Smulders^1^, Jean-Pierre Kaiser^2^, Stefano Zuin^3^, Kirsten L Van Landuyt^4^, Luana Golanski^5^, Peter Wick^2^, Peter HM Hoet^1,*^

**Affilations**

^1^ Laboratory of Pneumology, Unit for Lung Toxicology, KU Leuven, Leuven, Belgium

^2^ Empa, Swiss Federal Laboratories for Materials Science and Technology, Laboratory for Materials-Biology Interactions, CH-9014 St. Gallen, Switzerland

^3^ Venice Research Consortium, c/o VEGA Park - Venice Gateway for Science and Technology, Venice, Italy

^4^ KU Leuven BIOMAT, Department of Oral Health Sciences, KU Leuven, Leuven, Belgium

^5^ CEA-Grenoble, Liten, Laboratory of Tracer Technologies, France

**Fig. S1: TNF-α release of TLR4 reporter cells after exposure to different concentrations of endotoxin.**

TLR4 reporter cells were exposed to different concentrations of endotoxin (*E. coli* strain O111:B4) for 22h. TNF-α concentrations were measured in the supernatant using a TNF-α ELISA kit (Enzo Life Sciences, Antwerpen, Belgium).

**Fig. S2: Particle samples (20 mg/ml) after centrifugation (2 min, 1000g)**


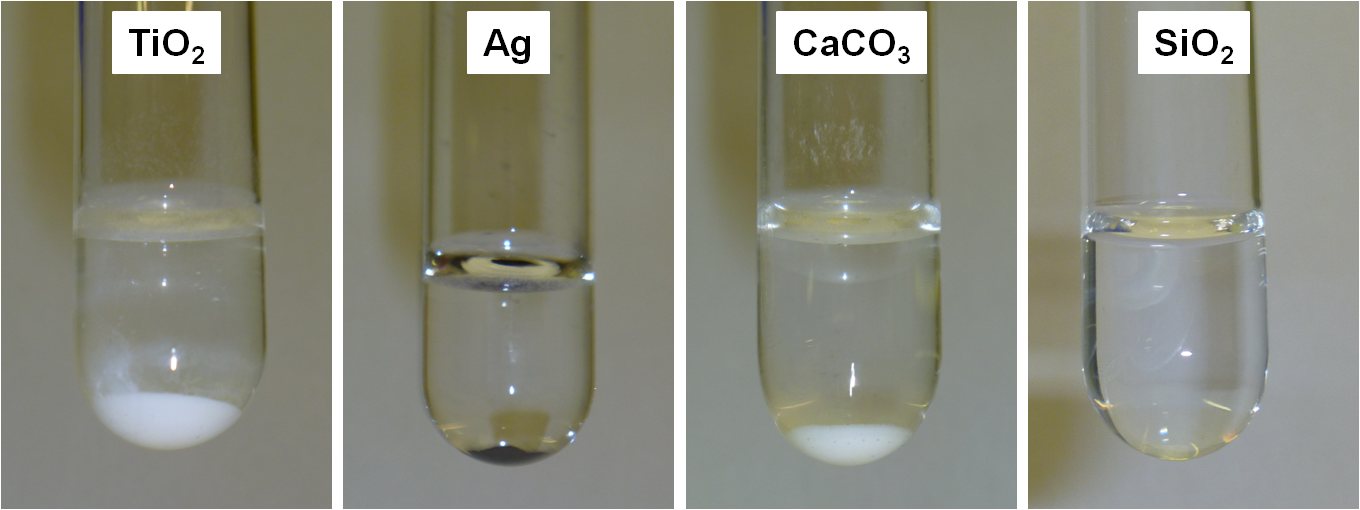

Supplement: Additional file 1 — Figure S1. TNF-α release of TLR4 reporter cells after exposure to different concentrations of endotoxin. Figure S2. Pictures of particle samples (20 mg/ml) after centrifugation (2 min, 1000 g). [file 1743-8977-9-41-S1.docx]
